# Supplementary material for: Escape from X inactivation is directly modulated by Xist noncoding RNA
Source: Nat Cell Biol. 2025 Dec 15;28(1):166–81. doi: 10.1038/s41556-025-01823-6 (PMC12807875; doi:10.1038/s41556-025-01823-6)
Supplement: Supplementary file 1 — Supplementary Figs. 1 and 2. [file 41556_2025_1823_MOESM1_ESM.pdf]

nature cell biology

Supplementary information https://doi.org/10.1038/s41556-025-01823-6

Escape from X inactivation is directly modulated by Xist noncoding RNA

In the format provided by the authors and unedited

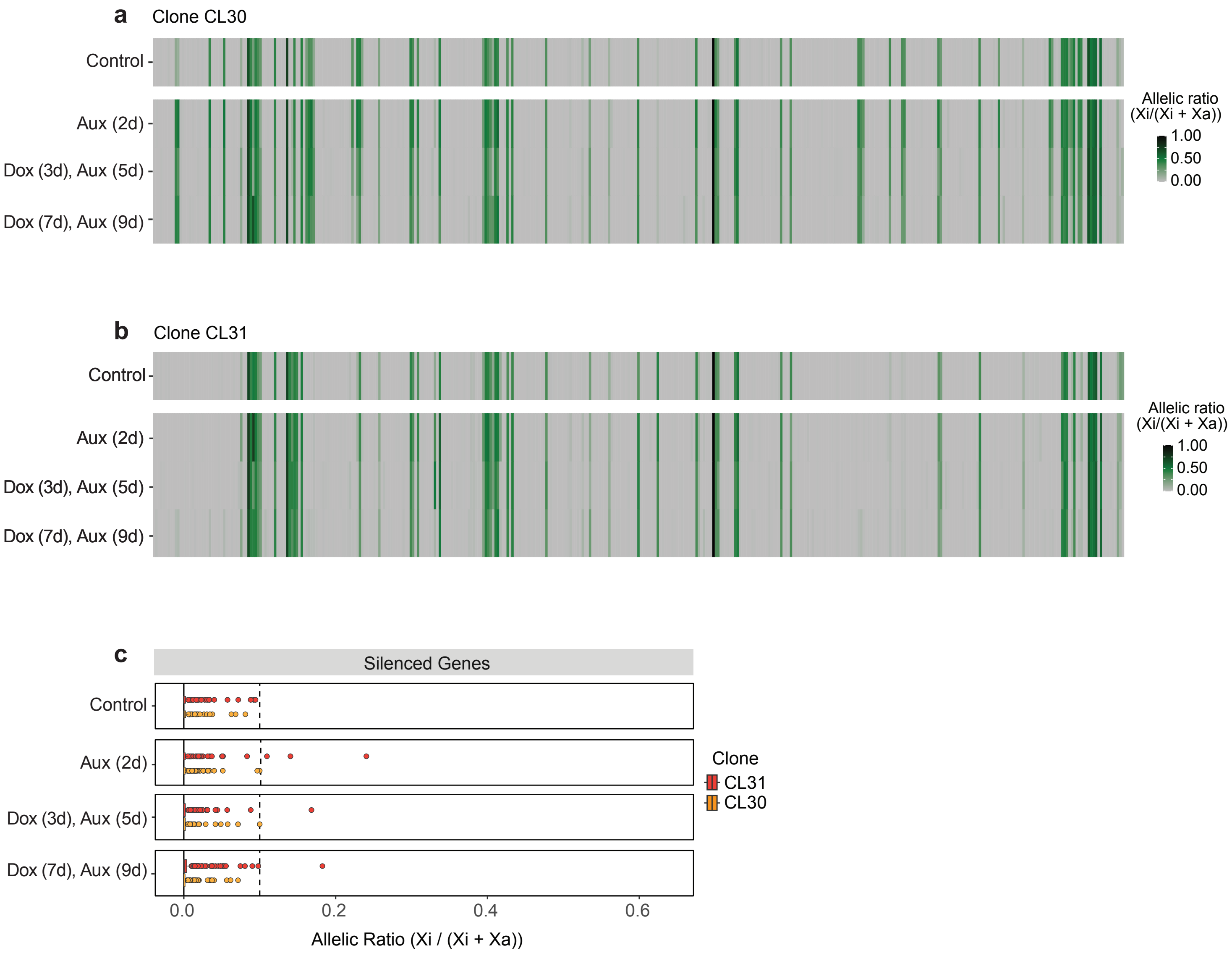

Supplementary Figure 1

a, b: Heatmap showing allelic ratios of X-linked genes in untreated condition and upon Xist for 3 days (Dox (3d)) and 7 days (Dox (7d)) and in combination with Auxin treatment (Dox (3d), Aux (5d) and Dox (7d), Aux (9d)) in NPC clone CL30 **(a)** and CL3 **(b)**. Shown are average values across replicates (Control: n = 3, Aux (2d): n = 2, Dox (3d), Aux (5d): n = 2, Dox (7d), Aux (9d): n = 2). **c,** Boxplots showing allelic ratios of X-inactivated genes (average allelic ratio across control replicates < 0.1) in NPC clones CL30 and CL31. Three genes show reactivation after 2 days of auxin treatment, 1 gene after Dox (3d), Aux (5d), and 1 gene after Dox (7d), Aux (9d). Box plots show median, 25- and 75-quantiles and 1.5x inter-quartile range.

ATGCCAAAGAGACCCAGACCCTCTAGATTAGATAAAAGTAAAGTGATTAACAGCGCATTAGAGCTG
CTTAATGAGGTCGGAATCGAAGGTTTAACAACCCGTAAACTCGCCCAGAAGCTTGGTGTAGAGCAG
CCTACACTGTATTGGCATGTAAAAAATAAGCGGGCTTTGCTCGACGCCTTAGCCATTGAGATGTTAG
ATAGGCACCATACTCACTTTTGCCCTTTAAAAGGGGAAAGCTGGCAAGATTTTTTACGCAATAACGC
TAAAAGTTTTAGATGTGCTTTACTAAGTCATCGCAATGGAGCAAAAGTACATTCAGATACACGGCCTA
CAGAAAAACAGTATGAAACTCTCGAAAATCAATTAGCCTTTTTATGCCAACAAGGTTTTTCACTAGAG
AACGCGTTATATGCACTCAGCGCTGTGGGGCATTTTACTTTAGGTTGCGTATTGGAAGATCAAGAGC
ATCAAGTCGCTAAAGAAGAAAGGGAAACACCTACTACTGATAGTATGCCGCCATTATTACGACAAG
CTATCGAATTATTTGATCACCAAGGTGCAGAGCCAGCCTTCTTATTCGGCCTTGAATTGATCATATGC
GGATTAGAAAAACAACTTAAATGTGAAAGTGGGTCCGCGTACAGCCGCGCGCGTACGAAAAACAA
TTACGGGTCTACCATCGAGGGCCTGCTCGATCTCCCGGACGACGACGCCCCCGAAGAGGCGGG
GCTGGCGGCTCCGCGCCTGTCCTTTCTCCCCGCGGGACACACGCGCAGACTGTCGACGGCCC
CCCCGACCGATGTCAGCCTGGGGGACGAGCTCCACTTAGACGGCGAGGACGTGGCGATGGCG
CATGCCGACGCGCTAGACGATTTCGATCTGGACATGTTGGGGGACGGGGATTCCCCGGGTCCGG
GATTTACCCCCCACGACTCCGCCCCCTACGGCGCTCTGGATATGGCCGACTTCGAGTTTGAGCA
GATGTTTACCGATGCCCTTGGAATTGACGAGTACGGTGGGTAG

Supplementary Figure 2
Sequence of the rtTA transactivator integrated at the Rosa26 locus in TX mice
