# Supplementary material for: Escape from X inactivation is directly modulated by Xist noncoding RNA
Source: Nat Cell Biol. 2025 Dec 15;28(1):166–81. doi: 10.1038/s41556-025-01823-6 (PMC12807875; doi:10.1038/s41556-025-01823-6)
Supplement: Supplementary file 2 — Reporting Summary [file 41556_2025_1823_MOESM2_ESM.pdf]

nature portfolio

Corresponding author(s): Edith Heard
Agnese Loda

Last updated by author(s): Oct 8, 2025

Reporting Summary

Nature Portfolio wishes to improve the reproducibility of the work that we publish. This form provides structure for consistency and transparency in reporting. For further information on Nature Portfolio policies, see our Editorial Policies and the Editorial Policy Checklist.

Statistics

For all statistical analyses, confirm that the following items are present in the figure legend, table legend, main text, or Methods section.

n/a	Confirmed	
☐	☒	The exact sample size (*n*) for each experimental group/condition, given as a discrete number and unit of measurement
☒	☐	A statement on whether measurements were taken from distinct samples or whether the same sample was measured repeatedly
☐	☒	The statistical test(s) used AND whether they are one- or two-sided<br>*Only common tests should be described solely by name; describe more complex techniques in the Methods section.*
☐	☒	A description of all covariates tested
☐	☒	A description of any assumptions or corrections, such as tests of normality and adjustment for multiple comparisons
☐	☒	A full description of the statistical parameters including central tendency (e.g. means) or other basic estimates (e.g. regression coefficient) AND variation (e.g. standard deviation) or associated estimates of uncertainty (e.g. confidence intervals)
☐	☒	For null hypothesis testing, the test statistic (e.g. *F*, *t*, *r*) with confidence intervals, effect sizes, degrees of freedom and *P* value noted<br>*Give P values as exact values whenever suitable.*
☒	☐	For Bayesian analysis, information on the choice of priors and Markov chain Monte Carlo settings
☒	☐	For hierarchical and complex designs, identification of the appropriate level for tests and full reporting of outcomes
☐	☒	Estimates of effect sizes (e.g. Cohen's *d*, Pearson's *r*), indicating how they were calculated

Our web collection on statistics for biologists contains articles on many of the points above.

Software and code

Policy information about availability of computer code

Data collection	No software was used for data collection.
Data analysis	All code to reproduce the analysis presented in the paper is available on github in the repository https://github.com/odomlab2/xist_project. The preprocessing workflows for RNA-Seq and CUT&RUN data are available at the following links: https://github.com/yuviaapr/allele-specific_RNA-seq and https://github.com/yuviaapr/allele-specific_CUTandRUN.<br><br>Published software used: trim_galore (v0.6.6, v0.6.7, v0.6.3), Picard Tools (v2.20.8), MACS2 (v2.2.7.1), bowtie2 (v2.3.4.1), SNPsplit (SNPsplit_genome_preparation script, v0.3.4, v0.5.0), STAR (v2.5.3a, v2.7.2b), featureCounts (v2.0.1), stats, v4.2.0, DESeq2 (R, v1.36.0, v1.38.3, v.1.44.0), EnsDb.Mmusculus.v79, (v2.99.0), cooler (v0.8.9), cooltools (v0.3.2), pyGenomeTracks, DiffBind (v3.8.4),Nextflow (20.04.1), BSgenome.Mmusculus.UCSC.mm10, ChipSeeker (v1.34.1), TxDb.Mmusculus.UCSC.mm10.knownGene (v3.10.0), scran (v1.24.1), methylseq nextflow pipeline (v2.3.0, v3.0.0, NextFlow v22.10.6,v24.10.4), Hi-C Pro pipeline (v2.11.4), FastQC (v0.11.8, v0.11.9), samtools (v1.9), Bismark (v0.23.1), deeptools (v3.5.1, v3.5.2), HiCExplorer (v3.7.2), Fiji/ImageJ software.

For manuscripts utilizing custom algorithms or software that are central to the research but not yet described in published literature, software must be made available to editors and reviewers. We strongly encourage code deposition in a community repository (e.g. GitHub). See the Nature Portfolio guidelines for submitting code & software for further information.

Data

Policy information about availability of data

All manuscripts must include a data availability statement. This statement should provide the following information, where applicable:

- Accession codes, unique identifiers, or web links for publicly available datasets
- A description of any restrictions on data availability
- For clinical datasets or third party data, please ensure that the statement adheres to our policy

All newly generated data was deposted in the Gene Expression Omnibus (GEO) database, under the accession number GSE259400.

Research involving human participants, their data, or biological material

Policy information about studies with human participants or human data. See also policy information about sex, gender (identity/presentation), and sexual orientation and race, ethnicity and racism.

Reporting on sex and gender	This research does not involve human participants, their data, or biological material.
Reporting on race, ethnicity, or other socially relevant groupings	This research does not involve human participants, their data, or biological material.
Population characteristics	This research does not involve human participants, their data, or biological material.
Recruitment	This research does not involve human participants, their data, or biological material.
Ethics oversight	This research does not involve human participants, their data, or biological material.

Note that full information on the approval of the study protocol must also be provided in the manuscript.

Field-specific reporting

Please select the one below that is the best fit for your research. If you are not sure, read the appropriate sections before making your selection.

☒ Life sciences ☐ Behavioural & social sciences ☐ Ecological, evolutionary & environmental sciences

For a reference copy of the document with all sections, see nature.com/documents/nr-reporting-summary-flat.pdf

Life sciences study design

All studies must disclose on these points even when the disclosure is negative.

Sample size	No statistical analyses were used to predetermine sample sizes. Adequate statistics has been applied throughout the manuscript in order to make sure that the observed effects are significant given the reported sample size
Data exclusions	No data was excluded
Replication	Experiments were independently repeated and the number of replicates is indicated in the manuscript
Randomization	Samples were not randomized, given that samples were grouped according to their respective genotypes.
Blinding	No blinding was performed during collection or data analysis.

Reporting for specific materials, systems and methods

We require information from authors about some types of materials, experimental systems and methods used in many studies. Here, indicate whether each material, system or method listed is relevant to your study. If you are not sure if a list item applies to your research, read the appropriate section before selecting a response.

Materials & experimental systems

n/a	Involved in the study
☐	☒ Antibodies
☐	☒ Eukaryotic cell lines
☒	☐ Palaeontology and archaeology
☐	☒ Animals and other organisms
☒	☐ Clinical data
☒	☐ Dual use research of concern
☒	☐ Plants

Methods

n/a	Involved in the study
☒	☐ ChIP-seq
☒	☐ Flow cytometry
☒	☐ MRI-based neuroimaging

Antibodies

Antibodies used	anti-GFAP (#173002, Synaptic System, dilution: 1:400) , anti-Ki67 (#556003, BD biosciences, dilution: 1:200), anti-H3K27me3 (#9733, Cell Signaling, dilution: 1:100), anti-H2AK119ubi (#D27C4, Cell Signaling, dilution: 1:100)
Validation	All commercially available antibodies were validated by the manufacturers.

Eukaryotic cell lines

Policy information about cell lines and Sex and Gender in Research

Cell line source(s)	TX1072 female mouse embryonic stem cells (mESCs) used in this study were derived from a cross of a TX/TX R26rtTA/rtTA female (Savarese 2006) with a Mus musculus castaneus male according to the animal care guidelines of Institut Curie (Paris) as described in Schulz 2014. NPC lines were generated upon in vitro differentiation of this ESC line. Astrocytes were generated upon in vitro differentiation of the NPC line E6, derived from TX1072 ESCs.
Authentication	TX1072 ESCs were not authenticated. Next Generation Sequencing confirmed their identity as mouse cells
Mycoplasma contamination	All cell lines used in this study were tested negative for mycoplasma contamination.
Commonly misidentified lines (See ICLAC register)	N/A

Animals and other research organisms

Policy information about studies involving animals; ARRIVE guidelines recommended for reporting animal research, and Sex and Gender in Research

Laboratory animals	Female pre-implantation embryos and E8.5 extraembryonic tissues analyzed in this study were derived from natural mating between 8 to 40 week-old C57BL/6 TX males Xptet/Y; R26rtTA/WT or Xptet/Y; R26rtTA/rtTA (Savarese 2006) with wild type Mus musculus molossinus (JF1/Ms) females. 5 to 7 week-old and 8 to 10 week-old females were used for superovulation and natural mating, respectively. Mice were housed under a 12h light/dark cycle (light from 07:00 to 19:00), with ad libitum access to food and beverages. Temperatures of 18C to 23C, 40-60% humidity.
Wild animals	The study did not involved wild animals.
Reporting on sex	We crossed female and male mice to obtain pre-implantation embryos or E8.5 embryos. As our work focuses on X-chromosome inactivation, which is a female-specific process, we only analyzed female embryos. We determined the sex of the embryos by PCR.
Field-collected samples	This study did not involve samples collected from the field.
Ethics oversight	All experimental designs and procedures were performed in agreement with the rules and regulations of the Institutional Animal Care and Use Committee (IACUC) of the European Molecular Biology Laboratory (EMBL) under protocols number 019-03-21EH and 24-007_HD_EH.

Note that full information on the approval of the study protocol must also be provided in the manuscript.

Plants

Seed stocks	*Report on the source of all seed stocks or other plant material used. If applicable, state the seed stock centre and catalogue number. If plant specimens were collected from the field, describe the collection location, date and sampling procedures.*
Novel plant genotypes	*Describe the methods by which all novel plant genotypes were produced. This includes those generated by transgenic approaches, gene editing, chemical/radiation-based mutagenesis and hybridization. For transgenic lines, describe the transformation method, the number of independent lines analyzed and the generation upon which experiments were performed. For gene-edited lines, describe the editor used, the endogenous sequence targeted for editing, the targeting guide RNA sequence (if applicable) and how the editor was applied.*
Authentication	*Describe any authentication procedures for each seed stock used or novel genotype generated. Describe any experiments used to assess the effect of a mutation and, where applicable, how potential secondary effects (e.g. second site T-DNA insertions, mosiacism, off-target gene editing) were examined.*
